# An Epidemiological Network Model for Disease Outbreak Detection

**DOI:** 10.1371/journal.pmed.0040210

**Published:** 2007-06-26

**Authors:** Ben Y Reis, Isaac S Kohane, Kenneth D Mandl

**Affiliations:** Children's Hospital Informatics Program, Harvard-MIT Division of Health Sciences and Technology, Children's Hospital, Harvard Medical School, Boston, Massachusetts, United States of America; University of Texas at Austin, United States of America

## Abstract

**Background:**

Advanced disease-surveillance systems have been deployed worldwide to provide early detection of infectious disease outbreaks and bioterrorist attacks. New methods that improve the overall detection capabilities of these systems can have a broad practical impact. Furthermore, most current generation surveillance systems are vulnerable to dramatic and unpredictable shifts in the health-care data that they monitor. These shifts can occur during major public events, such as the Olympics, as a result of population surges and public closures. Shifts can also occur during epidemics and pandemics as a result of quarantines, the worried-well flooding emergency departments or, conversely, the public staying away from hospitals for fear of nosocomial infection. Most surveillance systems are not robust to such shifts in health-care utilization, either because they do not adjust baselines and alert-thresholds to new utilization levels, or because the utilization shifts themselves may trigger an alarm. As a result, public-health crises and major public events threaten to undermine health-surveillance systems at the very times they are needed most.

**Methods and Findings:**

To address this challenge, we introduce a class of epidemiological network models that monitor the relationships among different health-care data streams instead of monitoring the data streams themselves. By extracting the extra information present in the relationships between the data streams, these models have the potential to improve the detection capabilities of a system. Furthermore, the models' relational nature has the potential to increase a system's robustness to unpredictable baseline shifts. We implemented these models and evaluated their effectiveness using historical emergency department data from five hospitals in a single metropolitan area, recorded over a period of 4.5 y by the Automated Epidemiological Geotemporal Integrated Surveillance real-time public health–surveillance system, developed by the Children's Hospital Informatics Program at the Harvard-MIT Division of Health Sciences and Technology on behalf of the Massachusetts Department of Public Health. We performed experiments with semi-synthetic outbreaks of different magnitudes and simulated baseline shifts of different types and magnitudes. The results show that the network models provide better detection of localized outbreaks, and greater robustness to unpredictable shifts than a reference time-series modeling approach.

**Conclusions:**

The integrated network models of epidemiological data streams and their interrelationships have the potential to improve current surveillance efforts, providing better localized outbreak detection under normal circumstances, as well as more robust performance in the face of shifts in health-care utilization during epidemics and major public events.

## Abstract 2

Most surveillance systems are not robust to shifts in health care utilization. Ben Reis and colleagues developed network models that detected localized outbreaks better and were more robust to unpredictable shifts.

## Editors' Summary

**Background.:**

The main task of public-health officials is to promote health in communities around the world. To do this, they need to monitor human health continually, so that any outbreaks (epidemics) of infectious diseases (particularly global epidemics or pandemics) or any bioterrorist attacks can be detected and dealt with quickly. In recent years, advanced disease-surveillance systems have been introduced that analyze data on hospital visits, purchases of drugs, and the use of laboratory tests to look for tell-tale signs of disease outbreaks. These surveillance systems work by comparing current data on the use of health-care resources with historical data or by identifying sudden increases in the use of these resources. So, for example, more doctors asking for tests for salmonella than in the past might presage an outbreak of food poisoning, and a sudden rise in people buying over-the-counter flu remedies might indicate the start of an influenza pandemic.

**Why Was This Study Done?:**

Existing disease-surveillance systems don't always detect disease outbreaks, particularly in situations where there are shifts in the baseline patterns of health-care use. For example, during an epidemic, people might stay away from hospitals because of the fear of becoming infected, whereas after a suspected bioterrorist attack with an infectious agent, hospitals might be flooded with “worried well” (healthy people who think they have been exposed to the agent). Baseline shifts like these might prevent the detection of increased illness caused by the epidemic or the bioterrorist attack. Localized population surges associated with major public events (for example, the Olympics) are also likely to reduce the ability of existing surveillance systems to detect infectious disease outbreaks. In this study, the researchers developed a new class of surveillance systems called “epidemiological network models.” These systems aim to improve the detection of disease outbreaks by monitoring fluctuations in the relationships between information detailing the use of various health-care resources over time (data streams).

**What Did the Researchers Do and Find?:**

The researchers used data collected over a 3-y period from five Boston hospitals on visits for respiratory (breathing) problems and for gastrointestinal (stomach and gut) problems, and on total visits (15 data streams in total), to construct a network model that included all the possible pair-wise comparisons between the data streams. They tested this model by comparing its ability to detect simulated disease outbreaks implanted into data collected over an additional year with that of a reference model based on individual data streams. The network approach, they report, was better at detecting localized outbreaks of respiratory and gastrointestinal disease than the reference approach. To investigate how well the network model dealt with baseline shifts in the use of health-care resources, the researchers then added in a large population surge. The detection performance of the reference model decreased in this test, but the performance of the complete network model and of models that included relationships between only some of the data streams remained stable. Finally, the researchers tested what would happen in a situation where there were large numbers of “worried well.” Again, the network models detected disease outbreaks consistently better than the reference model.

**What Do These Findings Mean?:**

These findings suggest that epidemiological network systems that monitor the relationships between health-care resource-utilization data streams might detect disease outbreaks better than current systems under normal conditions and might be less affected by unpredictable shifts in the baseline data. However, because the tests of the new class of surveillance system reported here used simulated infectious disease outbreaks and baseline shifts, the network models may behave differently in real-life situations or if built using data from other hospitals. Nevertheless, these findings strongly suggest that public-health officials, provided they have sufficient computer power at their disposal, might improve their ability to detect disease outbreaks by using epidemiological network systems alongside their current disease-surveillance systems.

**Additional Information.:**

Please access these Web sites via the online version of this summary at http://dx.doi.org/10.1371/journal.pmed.0040210.

- Wikipedia pages on public health (note that Wikipedia is a free online encyclopedia that anyone can edit, and is available in several languages)
- A brief description from the World Health Organization of public-health surveillance (in English, French, Spanish, Russian, Arabic, and Chinese)
- A detailed report from the US Centers for Disease Control and Prevention called “Framework for Evaluating Public Health Surveillance Systems for the Early Detection of Outbreaks”
- The International Society for Disease Surveillance Web site

## Introduction

Understanding and monitoring large-scale disease patterns is critical for planning and directing public-health responses during pandemics [1–5]. In order to address the growing threats of global infectious disease pandemics such as influenza [6], severe acute respiratory syndrome (SARS) [7], and bioterrorism [8], advanced disease-surveillance systems have been deployed worldwide to monitor epidemiological data such as hospital visits [9,10], pharmaceutical orders [11], and laboratory tests [12]. Improving the overall detection capabilities of these systems can have a wide practical impact. Furthermore, it would be beneficial to reduce the vulnerability of many of these systems to shifts in health-care utilization that can occur during public-health emergencies such as epidemics and pandemics [13–15] or during major public events [16].

We need to be prepared for the shifts in health-care utilization that often accompany major public events, such as the Olympics, caused by population surges or closures of certain areas to the public [16]. First, we need to be prepared for drops in health-care utilization under emergency conditions, including epidemics and pandemics where the public may stay away from hospitals for fear of being infected, as 66.7% reported doing so during the SARS epidemic in Hong Kong [13]. Similarly, a detailed study of the Greater Toronto Area found major drops in numerous types of health-care utilization during the SARS epidemic, including emergency department visits, physician visits, inpatient and outpatient procedures, and outpatient diagnostic tests [14]. Second, the “worried-well”—those wrongly suspecting that they have been infected—may proceed to flood hospitals, not only stressing the clinical resources, but also dramatically shifting the baseline from its historical pattern, potentially obscuring a real signal [15]. Third, public-health interventions such as closures, quarantines, and travel restrictions can cause major changes in health-care utilization patterns.

Such shifts threaten to undermine disease-surveillance systems at the very times they are needed most. During major public events, the risks and potential costs of bioterrorist attacks and other public-health emergencies increase. During epidemics, as health resources are already stretched, it is important to maintain disease outbreak–surveillance capabilities and situational awareness [4,5]. At present, many disease-surveillance systems rely either on comparing current counts with historical time-series models, or on identifying sudden increases in utilization (e.g., cumulative sum [CUSUM] or exponential weighted moving average [EWMA] [9,10]). These approaches are not robust to major shifts in health-care utilization: systems based on historical time-series models of health-care counts do not adjust their baselines and alert-thresholds to the new unknown utilization levels, while systems based on identifying sudden increases in utilization may be falsely triggered by the utilization shifts themselves.

In order to both improve overall detection performance and reduce vulnerability to baseline shifts, we introduce a general class of epidemiological network models that explicitly capture the relationships among epidemiological data streams. In this approach, the surveillance task is transformed from one of monitoring health-care data streams, to one of monitoring the *relationships* among these data streams: an epidemiological network begins with historical time-series models of the ratios between each possible pair of data streams being monitored. (As described in Discussion, it may be desirable to model only a selected subset of these ratios.) These ratios do not remain at a constant value; rather, we assume that these ratios vary in a predictable way according to seasonal and other patterns that can be modeled. The ratios predicted by these historical models are compared with the ratios observed in the actual data in order to determine whether an aberration has occurred. The complete approach is described in detail below.

These network models have two primary benefits. First, they take advantage of the extra information present in the relationships between the monitored data streams in order to increase overall detection performance. Second, their relational nature makes them more robust to the unpredictable shifts described above, as illustrated by the following scenario. The Olympics bring a large influx of people into a metropolitan area for 2 wk and cause a broad surge in overall health-care utilization. In the midst of this surge, a localized infectious disease outbreak takes place. The surge in overall utilization falsely triggers the alarms of standard biosurveillance models and thus masks the actual outbreak. On the other hand, since the surge affects multiple data streams similarly, the relationships between the various data streams are not affected as much by the surge. Since the network model monitors these relationships, it is able to ignore the surge and thus detect the outbreak.

Our assumption is that broad utilization shifts would affect multiple data streams in a similar way, and would thus not significantly affect the ratios among these data streams. In order to validate this assumption, we need to study the stability of the ratios around real-world surges. This assessment is difficult to do, since for most planned events, such as the Olympics, additional temporary health-care facilities are set up at the site of the event in order to deal with the expected surge. This preparation reduces or eliminates the surge that is recorded by the permanent health-care system, and therefore makes it hard to find data that describe surges. However, some modest shifts do appear in the health-care utilization data, and they are informative. We obtained data on the 2000 Sydney Summer Olympics directly from the Centre for Epidemiology and Research, New South Wales Department of Health, New South Wales Emergency Department Data Collection. The data show a 5% surge in visits during the Olympics. While the magnitude of this shift is far less dramatic than those expected in a disaster, the Sydney Olympics nonetheless provide an opportunity to measure the stability of the ratios under surge conditions. Despite the surge, the relative rates of major syndromic groups remained very stable between the same periods in 1999 and 2000. Injury visits accounted for 21.50% of overall visits in 1999, compared with an almost identical 21.53% in 2000. Gastroenteritis visits accounted for 5.84% in 1999, compared with 5.75% in 2000. As shown in Table 1, the resulting ratios among the different syndromic groups remained stable. Although we would have liked to examine the stability of ratios in the face of a larger surge, we were not able to find a larger surge for which multi-year health-care utilization data were available. It is important to note that while the above data about a planned event are informative, surveillance systems need to be prepared for the much larger surges that would likely accompany unplanned events, such as pandemics, natural disasters, or other unexpected events that cause large shifts in utilization.

**Table 1:**
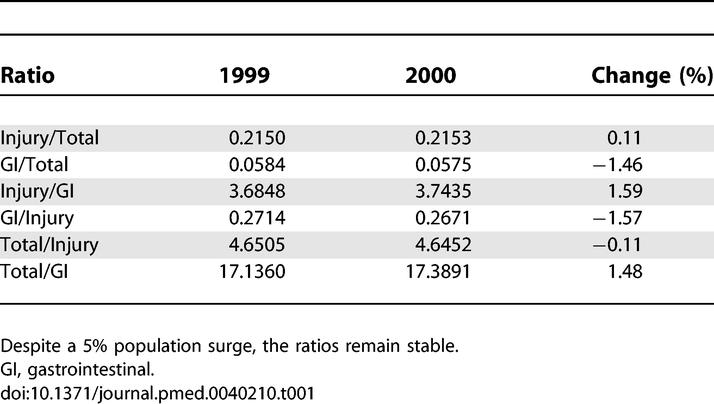
Stability of Ratios between Different Pairs of Syndromic Groups in Sydney in August 1999, and during the Same Period during the 2000 Summer Olympics

Initial motivation for this work originated as a result of the authors' experience advising the Hellenic Center for Infectious Diseases Control in advance of the 2004 Summer Olympics in Athens [17], where there was concern that a population surge caused by the influx of a large number of tourists would significantly alter health-care utilization patterns relative to the baseline levels recorded during the previous summer. The epidemiological network model was then formalized in the context of the US Centers for Disease Control and Prevention's nationwide BioSense health-surveillance system [18], for which the authors are researching improved surveillance methods for integration of inputs from multiple health-care data streams. BioSense collects and analyzes health-care utilization data, which have been made anonymous, from a number of national data sources, including the Department of Defense and the Veteran's Administration, and is now procuring local emergency department data sources from around the United States.

In order to evaluate the practical utility of this approach for surveillance, we constructed epidemiological network models based on real-world historical health-care data and compared their outbreak-detection performance to that of standard historical models. The models were evaluated using semi-synthetic data-streams—real background data with injected outbreaks—both under normal conditions and in the presence of different types of baseline shifts.

## Methods

### Data

The proposed epidemiological network model is compared with a previously described reference time-series model [19]. Both models are used to detect simulated outbreaks introduced into actual historical daily counts for respiratory-related visits, gastrointestinal-related visits, and total visits at five emergency departments in the same metropolitan area. The data cover a period of 1,619 d, or roughly 4.5 y. The first 1,214 d are used to train the models, while the final 405 d are used to test their performance.

The data are collected by the Automated Epidemiological Geotemporal Integrated Surveillance (AEGIS) real-time public health–surveillance system, developed by the Children's Hospital Informatics Program at the Harvard-MIT Division of Health Sciences and Technology on behalf of the Massachusetts Department of Public Health. AEGIS fully automates the monitoring of emergency departments across Massachusetts. The system receives automatic updates from the various health-care facilities and performs outbreak detection, alerting, and visualization functions for public-health personnel and clinicians. The AEGIS system incorporates both temporal and geospatial approaches for outbreak detection.

### Network Construction and Training

The goal of an epidemiological network model is to model the historical relationships among health-care data streams and to interpret newly observed data in the context of these modeled relationships. In the training phase, we construct time-series models of the ratios between all possible pairs of health-care utilization data streams. These models capture the weekly, seasonal, and long-term variations in these ratios. In the testing phase, the actual observed ratios are compared with the ratios predicted by the historical models.

We begin with *N* health-care data streams, *S_i_,* each describing daily counts of a particular syndrome category at a particular hospital. For this study, we use three syndromic categories (respiratory, gastrointestinal, and total visits) at five hospitals, for a total of *N* = 15 data streams. All possible pair-wise ratios are calculated among these *N* data streams, for a total of *N* ^2^ − *N* = 210 ratios, *R_ij_:* for each day, *t,* we calculate the ratio of the daily counts for stream *S_i_* to the daily counts for stream *S_j_.*

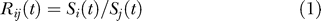



**Figure 1:**
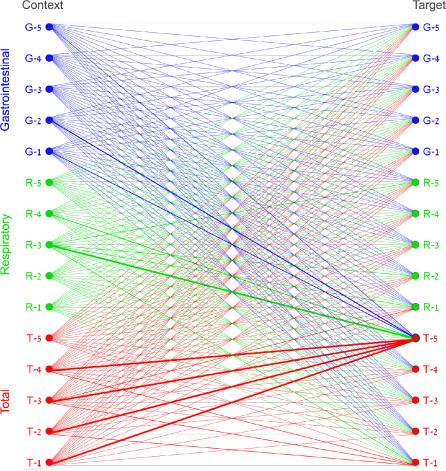
An Epidemiological Network Containing 15 Data Streams from Five Hospitals, Shown Responding to a Simulated Outbreak in Data Stream T-5 Each data stream appears twice in the network. The context nodes on the left are used for interpreting the activity of the target nodes on the right. Each edge represents the ratio of the target node divided by the context node, with a thicker edge indicating that the ratio is higher than expected.

For each ratio, the numerator *S_i_* is called the *target data stream,* and the denominator *S_j_* is called the *context data stream,* since the target data stream is said to be interpreted *in the context* of the context data stream, as described below. A sample epidemiological network consisting of 30 nodes and 210 edges is shown in Figure 1. The nodes in the network represent the data streams: each of the *N* data streams appears twice, once as a context data stream and another time as a target data stream. Edges represent ratios between data streams: namely, the target data stream divided by the context data stream.

To train the network, a time-series model, *R̄_ij_*, is fitted for each ratio, *R_ij_,* over the training period using established time-series methods [19]. The data are first smoothed with a 7-d exponential filter (EWMA with coefficient 0.5) to reduce the effects of noise [20]. The linear trend is calculated and subtracted out, then the overall mean is calculated and subtracted out, then the day-of-week means (seven values) are calculated and subtracted out, and finally the day-of-year means (365 values) are calculated. In order to generate predictions from this model, these four components are summed, using the appropriate values for day of the week, day of the year, and trend. The difference between each actual ratio, *R_ij_*, and its corresponding modeled prediction, *R̄_ij_*, is the error, *E_ij_*.

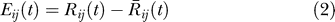



### Network Operation

During network operation, the goal of the network is to determine the extent to which the observed ratios among the data streams differ from the ratios predicted by the historical models. Observed ratios, *r_ij_,* are calculated from the observed data, and are compared with the expected ratios to yield the observed errors, *e_ij_:*

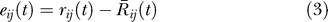



In order to interpret the magnitudes of these deviations from the expected values, the observed errors are compared with the historical errors from the training phase. A nonparametric approach is used to rank the current error against the historical errors. This rank is divided by the maximum rank (1 + the number of training days), resulting in a value of between 0 and 1, which is the individual aberration score, *w_ij_.*

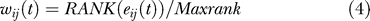



Conceptually, each of the individual aberration scores, *w_ij_,* represents the interpretation of the activity of the target data stream, *S_i_,* from the perspective of the activity at the context data stream, *S_j_:* if the observed ratio between these two data streams is exactly as predicted by the historical model, *e_ij_* is equal to 0 and *w_ij_* is equal to a moderate value. If the target data stream is higher than expected, *e_ij_* is positive and *w_ij_* is a higher value closer to 1. If it is lower than expected, *e_ij_* is positive and *w_ij_* is a lower value closer to 0. High aberration scores, *w_ij_,* are represented by thicker edges in the network visualization, as shown in Figure 1.

Some ratios are more unpredictable than others—i.e., they have a greater amount of variability that is not accounted for by the historical model, and thus a greater modeling error. The nonparametric approach to evaluating aberrations adjusts for this variability by interpreting a given aberration in the context of all previous aberrations for that particular ratio during the training period.

It is important to note that each individual aberration score, *w_ij_,* can be affected by the activities of both its target and context data streams. For example, it would be unclear from a single high *w_ij_* score as to whether the target data stream is unexpectedly high or whether the context data stream is unexpectedly low. In order to obtain an integrated consensus view of a particular target data stream, *S_i_,* an integrated consensus score, *c_i_,* is created by averaging together all the aberration scores that have *S_i_* as the target data stream (i.e., in the numerator of the ratio). This integrated score represents the collective interpretation of the activity at the target node, from the perspective of all the other nodes:

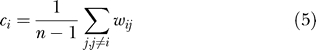
or

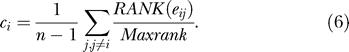



An alarm is generated whenever *c_i_* is greater than a threshold value *c_thresh_.* As described below, this threshold value is chosen to achieve a desired specificity. The nonparameteric nature of the individual aberration scores addresses the potential issue of outliers that would normally arise when taking an average. It is also important to note that while the integrated consensus score helps to reduce the effects of fluctuations in individual context data streams, it is still possible for an extreme drop in one context data stream to trigger a false alarm in a target data stream. This is particularly true in networks having few context data streams. In the case of only one context data stream, a substantial decrease in the count in the context data stream will trigger a false alarm in the target data stream.

### Reference Time-Series Model

For comparison, we also implement a reference time-series surveillance approach that models each health-care data stream directly, instead of modeling the relationships between data streams as above. This model uses the same time-series modeling methods described above and previously [19]. First, the daily counts data are smoothed with a 7-d exponential filter. The linear trend is calculated and subtracted out, then the overall mean is calculated and subtracted out, and then the mean for each day of the week (seven values) is calculated and subtracted out. Finally, the mean for each day of the year (365 values) is calculated and subtracted out. To generate a prediction, these four components are added together, taking the appropriate values depending on the particular day of the week and day of the year. The difference between the observed daily counts and the counts predicted by the model is the aberration score for that data stream. An alarm is generated whenever this aberration score is greater than a threshold value, chosen to achieve a desired level of specificity, as described below. By employing identical time-series methods for modeling the relationships between the streams in the network approach and modeling the actual data streams themselves in the reference approach, we are able to perform a controlled comparison between the two approaches.

### Simulated Outbreaks

**Figure 2:**
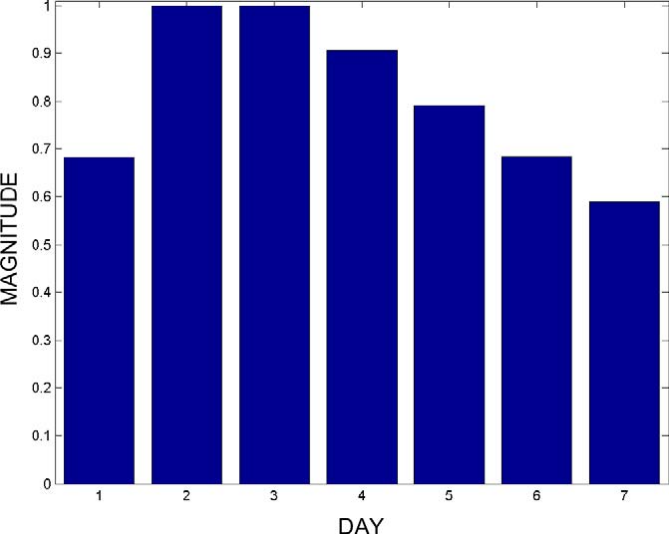
The Relative Magnitudes for each Day of the 7-d Outbreaks Used In the Simulations The magnitudes follow a lognormal outbreak curve.

Following established methods [19–21], we use semi-synthetic localized outbreaks to evaluate the disease-monitoring capabilities of the network. The injected outbreaks used here follow a 7-d lognormal temporal distribution (Figure 2), representing the epidemiological distribution of incubation times resulting from a single-source common vehicle infection, as described by Sartwell [22]. When injecting outbreaks into either respiratory- or gastrointestinal-related data streams, the same number of visits is also added to the appropriate total-visits data stream for that hospital in order to maintain consistency.

Multiple simulation experiments are performed, varying the number of data streams used in the network, the target data stream, *S_i_,* into which the outbreaks are introduced, and the magnitude of the outbreaks. While many additional outbreak types are possible, the simulated outbreaks used here serve as a paradigmatic set of benchmark stimuli for gauging the relative outbreak-detection performance of the different surveillance approaches.

## Results

### Network Performance

We constructed epidemiological networks from respiratory, gastrointestinal, and total daily visit data from five hospitals in a single metropolitan area, for a total of 15 data streams, *S_i_* (*N* = 15). In training the network, we modeled all possible pair-wise ratios between the 15 data streams, for a total of 210 ratios. For comparison, we implemented the reference time–series surveillance model described above, which uses the same time-series methods but instead of modeling the epidemiological relationships, models the 15 data streams directly. Semi-synthetic simulated outbreaks were used to evaluate the aberration-detection capabilities of the network, as described above. We simulated outbreaks across a range of magnitudes occurring at any one of the 15 data streams. For the first set of experiments, 486,000 tests were performed: 15 target data streams × 405 d of the testing period × 40 outbreak sizes (with a peak magnitude increase ranging from 2.5% to 100.0%) × two models (network versus reference). For the purposes of systematic comparison between the reference and network models, we allowed for the addition of fractional cases in the simulations.

We compared the detection sensitivities of the reference and network models by fixing specificity at a benchmark 95% and measuring the sensitivity of the model. In order to measure sensitivity at a desired specificity, we gradually increased the alarm threshold incrementally from 0 to the maximum value until the desired specificity was reached. We then measured the sensitivity at the same threshold. Sensitivity is defined in terms of outbreak-days—the proportion of all days during which outbreaks were occurring such that an alarm was generated. At 95% specificity, the network approach significantly outperformed the reference approach in detecting respiratory and gastrointestinal outbreaks, yielding 4.9% ± 1.9% and 6.0% ± 2.0% absolute increases in sensitivity, respectively (representing 19.1% and 34.1% relative improvements in sensitivity, respectively), for outbreaks characterized by a 37.5% increase on the peak day of the outbreak (Table 2). We found this ordering of sensitivities to be consistent over the range of outbreak sizes. For outbreaks introduced into the total-outbreak signals, the reference model achieved 2.1% ± 2% better absolute sensitivity than the network model (2.9% difference in relative sensitivity). This result is likely because the total-visit signals are much larger in absolute terms, and therefore the signal-to-noise ratio is higher (Table 3), making it easier for the reference model to detect the outbreaks. The “total outbreak” experiments were run for reasons of comprehensiveness, but it should be noted that there is no clear epidemiological correlate to an outbreak that affects all syndrome groups, other than a population surge, which the network models are designed to ignore as described in the discussion section. Also, an increase in total visits without an increase in respiratory or gastrointestinal visits may correspond to an outbreak in yet another syndrome category. Table 2 also shows results for the same experiments at three other practical specificity levels, and an average for all four specificity levels. In all cases, the network approach performs better for respiratory and gastrointestinal outbreaks and the reference model performs better in total-visit outbreaks.

**Table 2:**
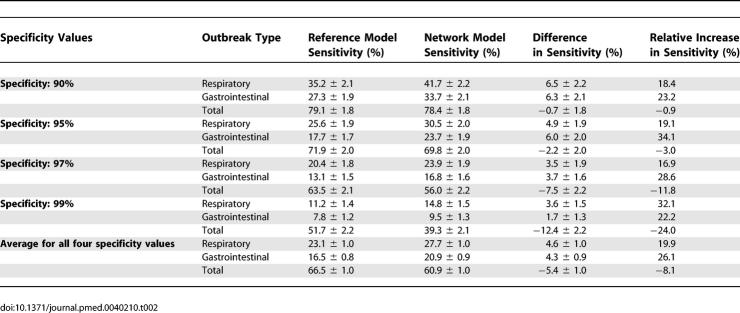
Average Sensitivity Comparisons for Network and Reference Models Measured at 90%, 95%, 97%, and 99% Specificity, and Averaged for all Four Specificity Values

**Table 3:**
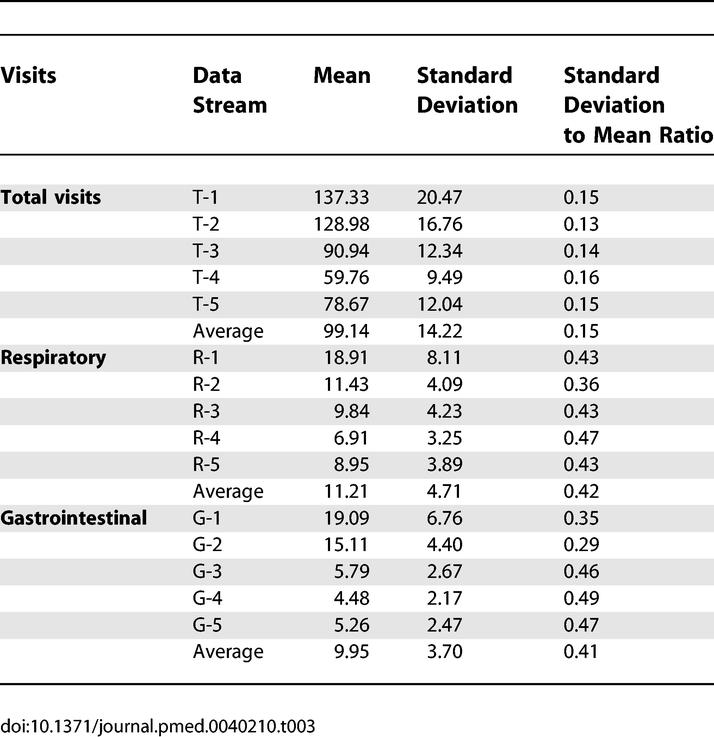
Basic Statistical Characterization of Data Streams

**Figure 3:**
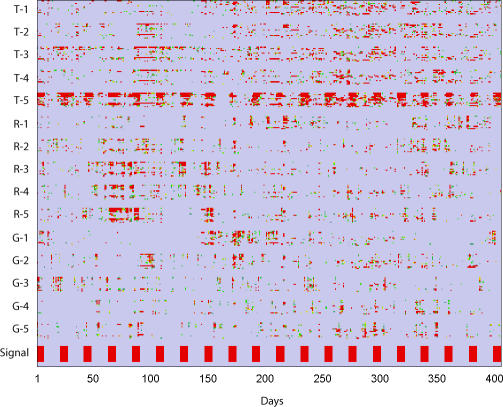
The Simulated Outbreaks Inserted at Periodic Intervals into Node T-5, Shown at Bottom of the Plot The responses of the 210 individual aberration scores are shown, grouped by target node.

**Figure 4:**
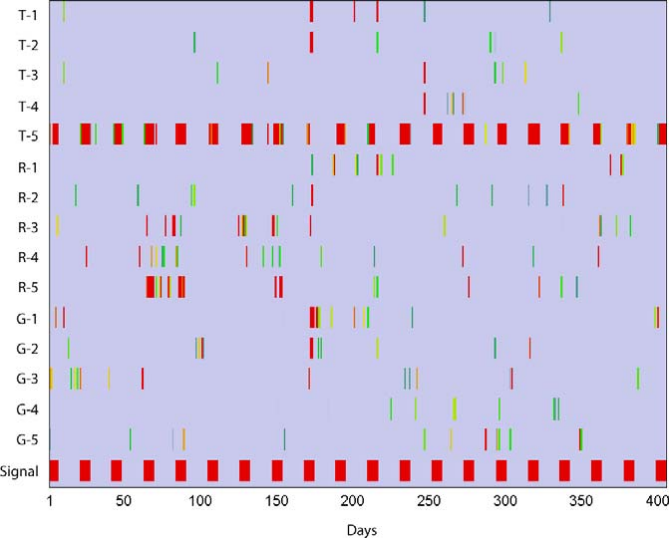
The Integrated Consensus Scores for each Target Data Stream The integrated consensus score for T-5 successfully reconstructs the simulated outbreaks presented to the system.

By visually inspecting the response of the network model to the outbreaks, it can be seen that while the individual aberration scores exhibited fairly noisy behavior throughout the testing period (Figure 3), the integrated consensus scores consolidated the information from the individual aberration scores, reconstructing the simulated outbreaks presented to the system (Figure 4).

### Effects of Network Composition

**Figure 5:**
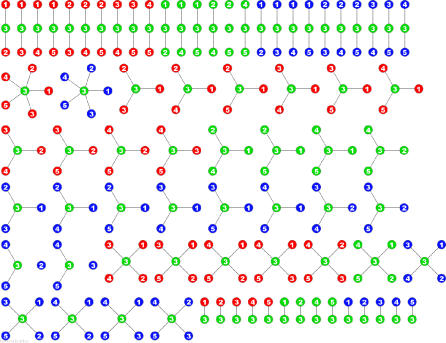
The 77 Possible Homogeneous Context Networks for Respiratory Target Data Stream R-3 Each network contains the target node plus a number of additional nodes of one type. In total, 1,155 networks are created and analyzed (15 target data streams × 77 networks). Red denotes total visits, green indicates respiratory, and blue indicates gastrointestinal.

Next, we studied the effects of different network compositions on detection performance, constructing networks of different sizes and constituent data streams (Figure 5). For each target data stream, we created 77 different homogeneous context networks—i.e., networks containing the target data stream plus between one and five additional data streams of a single syndromic category. In total, 1,155 networks were created and analyzed (15 target data streams × 77 networks). We then introduced simulated outbreaks characterized by a 37.5% increase in daily visit counts over the background counts in the target data stream on the peak day of the outbreak into the target data stream of each network, and calculated the sensitivity obtained from all the networks having particular size and membership characteristics, for a fixed benchmark specificity of 95%. In total, 467,775 tests were performed (1,155 networks × 405 d).

**Figure 6:**
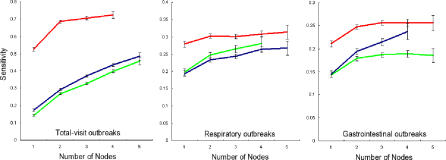
The Effects of Network Size and Membership on Detection Performance Each data point represents the sensitivity obtained from all the networks having particular size and membership characteristics, when presented with outbreaks in each of the five data streams for each target data stream type. In total, 467,775 tests are performed (1,155 networks × 405 d). Error bars shown are standard errors. Red denotes total visits, green indicates respiratory, and blue indicates gastrointestinal.

We found that detection performance generally increased with network size (Figure 6). Furthermore, regardless of which data stream contained the outbreaks, total-visit data streams provided the best context for detection. This is consistent with the greater statistical stability of the total-visits data streams, which on average had a far smaller variability (Table 3). Total data streams were also the easiest target data streams in which to detect outbreaks, followed by respiratory data streams, and then by gastrointestinal data streams. This result is likely because the number of injected cases is a constant proportion of stream size. For a constant number of injected cases, total data streams would likely be the hardest target data streams for detection.

**Figure 7:**
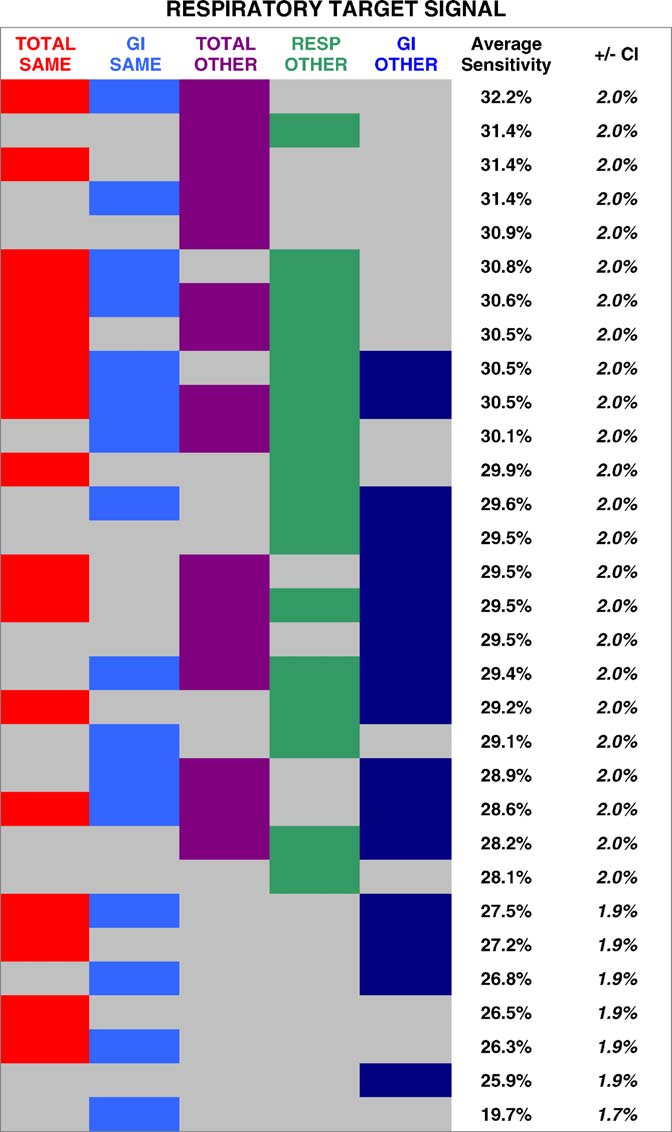
Systematic Comparison of Different Network Constructions: Respiratory Target Signals The average detection sensitivities are shown for 31 different context networks. Each column represents a different context group (total visits at the same hospital, gastrointestinal visits at the same hospital, total visits at all other hospitals, respiratory visits at all other hospitals, and gastrointestinal visits at other hospitals). Each row represents a different network construction. A cell is colored if the context group represented by that column was used in the network for that row. Rows are ranked by the average sensitivity achieved.

**Figure 8:**
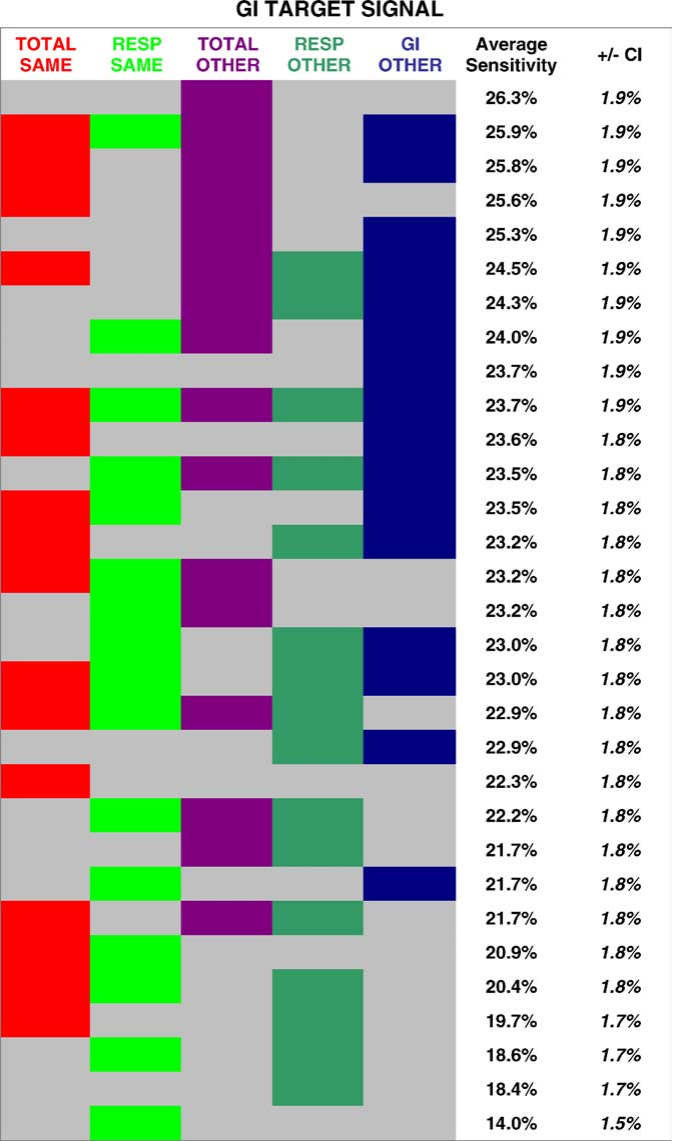
Systematic Comparison of Different Network Constructions: Gastrointestinal Target Signals The average detection sensitivities are shown for 31 different context networks. Each column represents a different context group (total visits at the same hospital, respiratory visits at the same hospital, total visits at all other hospitals, respiratory visits at all other hospitals, and gastrointestinal visits at other hospitals). Each row represents a different network construction. A cell is colored if the context group represented by that column was used in the network for that row. Rows are ranked by the average sensitivity achieved.

**Figure 9:**
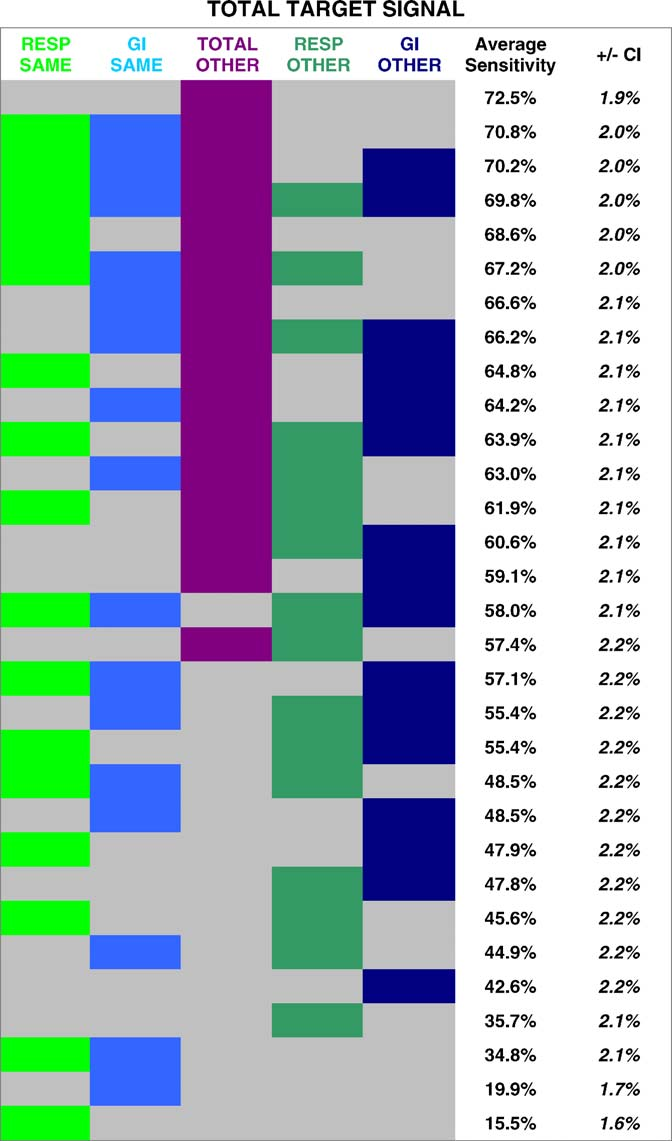
Systematic Comparison of Different Network Constructions: Total-Visit Target Signals The average detection sensitivities are shown for 31 different context networks. Each column represents a different context group (respiratory visits at the same hospital, gastrointestinal visits at the same hospital, total visits at all other hospitals, respiratory visits at all other hospitals, and gastrointestinal visits at other hospitals). Each row represents a different network construction. A cell is colored if the context group represented by that column was used in the network for that row. Rows are ranked by the average sensitivity achieved.

Next, we systematically compared the performance advantage gained from five key context groups. For a respiratory target signal, the five groups were as follows: (1) total visits at the same hospital; (2) total visits at all other hospitals; (3) gastrointestinal visits at the same hospital; (4) gastrointestinal visits at all other hospitals; and (5) respiratory visits at all other hospitals. If the target signal comprised gastrointestinal or total visits, the five context groups above would be changed accordingly, as detailed in Figures 7–9. Given the possibility of either including or excluding each of these five groups, there were 31 (2^5^ − 1) possible networks for each target signal.

The results of the above analysis are shown for respiratory (Figure 7), gastrointestinal (Figure 8), and total-visit target signals (Figure 9). Each row represents a different network construction. Rows are ranked by the average sensitivity achieved over the five possible target signals for that table. The following general trends are apparent. Total visits at all the other hospitals were the most helpful context group overall. Given a context of all the streams from the same hospital, it is beneficial to add total visits from other hospitals, as well as the same syndrome group from the other hospitals. Beginning with a context of total visits from the same hospital, there is a slight additional advantage in including a different syndrome group from the same hospital.

### Robustness to Epidemiological Shifts

In order to gauge the performance of the network and reference models in the face of baseline shifts in health-care utilization, we performed a further set of simulation experiments, where, in addition to the simulated outbreaks of peak magnitude 37.5%, we introduced various types and magnitudes of baseline shifts for a period of 200 d in the middle of the 405-d testing period. We compared the performance of the reference time-series model, the complete network model, and a network model containing only total-visit nodes. For respiratory and gastrointestinal outbreaks, we also compared the performance of a two-node network containing only the target data stream and the total-visit data stream from the same hospital.

**Figure 10:**
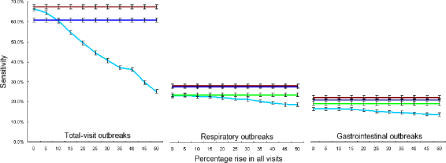
Simulation of a Population Surge during a Large Public Event To simulate a population surge during a large public event, all data streams are increased by a uniform amount (x-axis) for 200 d in the middle of the testing period. Full networks, total-visit networks, two-node networks (target data stream and total visits at the same hospital), and reference models are compared. Average results are shown for each target data stream type. Error bars are standard errors.

**Figure 11:**
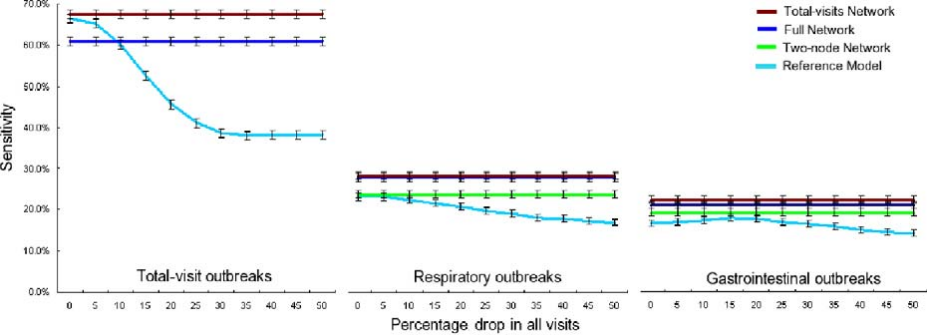
Simulation of a Frightened Public Staying Away from Hospitals during a Pandemic To simulate a frightened public staying away from hospitals during a pandemic, all data streams are dropped by a uniform amount (x-axis) for 200 d in the middle of the testing period. Full networks, total-visit networks, two-node networks (target data stream and total visits at the same hospital), and reference models are compared. Average results are shown for each target data stream type. Error bars are standard errors.

We began by simulating the effects of a large population surge, such as might be seen during a large public event. We did this by introducing a uniform increase across all data streams for 200 d in the middle of the testing period. We found that the detection performance of the reference model degraded rapidly with increasing baseline shifts, while the performance of the various network models remained stable (Figure 10). We next simulated the effects of a frightened public staying away from hospitals during an epidemic. We did this by introducing uniform drops across all data streams for 200 d. Here too, we found that the detection performance of the reference model degraded rapidly with increasing baseline shifts, while the performance of the various network models remained robust (Figure 11).

**Figure 12:**
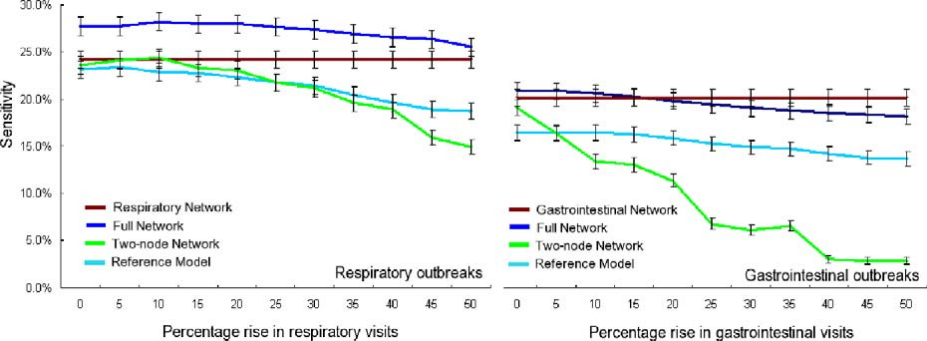
Simulation of the Effects of the Worried-Well Flooding Hospitals during a Pandemic To simulate the effects of the worried-well flooding hospitals during a pandemic, a targeted rise is introduced in only one type of data stream. Full networks, respiratory- or gastrointestinal-only networks, two-node networks, and reference models are compared. Error bars are standard errors.

We then simulated the effects of the “worried-well” on a surveillance system by introducing targeted increases in only one syndromic category—respiratory or gastrointestinal (Figure 12). We compared the performance of the reference model, a full-network model, the two-node networks described above, and a homogeneous network model containing only data streams of the same syndromic category as the target data stream. The performance of the full and homogeneous networks was superior to that of the reference model. The homogeneous networks, consisting of solely respiratory or gastrointestinal data streams, proved robust to the targeted shifts and achieved consistent detection performance even in the face of large shifts. This result is consistent with all the ratios in these networks being affected equally by the targeted baseline shifts. The performance of the full network degraded slightly in the face of larger shifts, while the performance of the two-node network degraded more severely. These results are because the two-node network did not include relationships that were unaffected by the shifts that could help stabilize performance. It should be noted that this same phenomenon—an increase in one syndromic category across multiple locations—may also be indicative of a widespread outbreak, as discussed further below.

## Discussion

In this paper, we describe an epidemiological network model that monitors the relationships between health-care utilization data streams for the purpose of detecting disease outbreaks. Results from simulation experiments show that these models deliver improved outbreak-detection performance under normal conditions compared with a standard reference time-series model. Furthermore, the network models are far more robust than the reference model to the unpredictable baseline shifts that may occur around epidemics or large public events. The results also show that epidemiological relationships are inherently valuable for surveillance: the activity at one hospital can be better understood by examining it in relation to the activity at other hospitals. In a previous paper [20], we showed the benefits of interpreting epidemiological data in its *temporal context*—namely, the epidemiological activity on surrounding days [23]. In the present study, we show that it is also beneficial to examine epidemiological data in its *network context*—i.e., the activity of related epidemiological data streams.

Based on the results obtained, it is clear that different types of networks are useful for detecting different types of signals. We present eight different classes of signals, their possible interpretations, and the approaches that would be able to detect them: the first four classes of signals involve increases in one or more data streams. (1) A rise in one syndrome group at a single location may correspond to a localized outbreak or simply a data irregularity. Such a signal could be detected by all network models as well as the reference model. (2) A rise in all syndrome groups at a single location probably corresponds to a geographical shift in utilization, (e.g., a quarantine elsewhere), as an outbreak would not be expected to cause an increase in all syndrome groups. Such a signal would be detected by network models that include multiple locations, and by the reference model. (3) A rise in one syndrome group across all locations may correspond to a widespread outbreak or may similarly result from the visits by the “worried-well.” Such a signal would be detected by network models that include multiple syndrome groups, and by the reference model. (4) A rise in all syndrome groups in all locations probably corresponds to a population surge, as an outbreak would not be expected to cause an increase in all syndrome groups. This signal would be ignored by all network models, but would be detected by the reference model.

The next four classes of signals involve decreases in one or more data streams. All of these signals are unlikely to be indicative of an outbreak, but are important for maintaining situational awareness in certain critical situations. As mentioned above, a significant decrease in a context data stream has the potential to trigger a false alarm in the target data stream, especially in networks with few context nodes. This is particularly true in two-node networks, where there is only one context data stream. (5) A fall in one syndrome group at a single location does not have an obvious interpretation. All models will ignore such a signal, since they are set to alarm on increases only. (6) A fall in all syndrome groups at a single location could represent a geographical shift in utilization (e.g., a local quarantine). All models will ignore such a signal. The baselines of all models will be affected, except for network models that include only nodes from single locations. (7) A fall in one syndrome group at all locations may represent a frightened public. All models will ignore such a signal. The baselines of all models will be affected, except for network models that include only nodes from single syndromic groups. (8) A fall in all data types at all locations may represent a regional population decrease or a frightened public staying away from hospitals out of concern for nosocomial infection (e.g., during an influenza pandemic). All models will ignore such a signal. The baseline of only the reference model will be affected. From this overview, it is clear that the network models are more robust than the reference model, with fewer false alarms (in scenarios 2 and 4) and less vulnerability to irregularities in baselines (in scenarios 6–8).

Based on the results obtained, when constructing epidemiological networks for monitoring a particular epidemiological data stream, we recommend prioritizing the inclusion of a total visits from all other hospitals, followed by total visits from the same hospital, followed by data streams of the same syndrome group from other hospitals and streams of different syndrome groups from the same hospital, followed by data streams of different syndrome groups from different hospitals. We further recommend that, in addition to full-network models, homogeneous network models (e.g., only respiratory nodes from multiple hospitals) be maintained for greater stability in the face of major targeted shifts in health-care utilization.

The two-node networks described above are similar in certain ways to the “rate”-based approach used by a small number of surveillance systems today [24–27]. Instead of monitoring daily counts directly, these systems monitor daily counts as a proportion of the total counts. For example, the respiratory-related visits at a certain hospital could be tracked as a percentage of the total number of visits to that hospital, or alternatively, as a percentage of the total number of respiratory visits in the region. These “rate”-based approaches have been proposed where absolute daily counts are too unstable for modeling [24], or where population-at-risk numbers are not available for use in spatiotemporal scan statistics [25]. The approach presented here is fundamentally different in that it explicitly models and tracks *all* possible inter-data stream relationships, not just those between a particular data stream and its corresponding total-visits data stream. Furthermore, the present approach is motivated by the desire to increase robustness in the face of large shifts in health-care utilization that may occur during epidemics or major public events. As such, this study includes a systematic study of the models' responses to different magnitudes of both broad and targeted baseline shifts. The two-node networks described above are an example of this general class of “rate”-based models. While the two-node approach works well under normal conditions, it is not as robust to targeted shifts in health-care utilization as larger network models. The results therefore show that there is value in modeling all, or a selected combination of the relationships among health-care data streams, not just the relationship between a data stream and its corresponding total-visits data stream.

Modeling all these relationships involves an order-*N* expansion of the number of models maintained internally by the system: *N* ^2^ − *N* models are used to monitor *N* data streams. The additional information inherent in this larger space is extracted to improve detection performance, after which the individual model outputs are collapsed back to form the *N* integrated outputs of the system. Since the number of models grows quadratically with the number of data streams, *N,* the method can become computationally intensive for large numbers of streams. In such a case, the number of models could be minimized by, for example, constructing only networks that include nodes from different syndrome groups but from the same hospital, or alternatively, including all context nodes from the same hospital and only total-visit nodes from other hospitals.

This work is different from other recent epidemiological research that has described simulated contact networks of individual people moving about in a regional environment and transmitting infectious diseases from one person to another. These simulations model the rate of spread of an infection under various conditions and interventions and help prepare for emergency scenarios by evaluating different health policies. On the other hand, we studied *relational* networks of hospitals monitoring health-care utilization in a regional environment, for the purpose of detecting localized outbreaks in a timely fashion and maintaining situational awareness under various conditions. Our work is also focused on generating an integrated network view of an entire health-care environment.

Limitations of this study include the use of simulated infectious disease outbreaks and baseline shifts. We use a realistic outbreak shape and baseline shift pattern, and perform simulation experiments varying the magnitudes of both of these. While other outbreak shapes and baseline shift patterns are possible, this approach allows us to create a paradigmatic set of conditions for evaluating the relative outbreak-detection performance of the various approaches [21]. Another possible limitation is that even though our findings are based on data across multiple disease categories (syndromes), multiple hospitals, and multiple years, relationships between epidemiological data streams may be different in other data environments. Also, our methods are focused on temporal modeling, and therefore do not have an explicit geospatial representation of patient location, even though grouping the data by hospital does preserve a certain degree of geospatial information. The specific temporal modeling approach used requires a solid base of historical data for the training set. However, this modeling approach is not integral to the network strategy, and one could build an operational network by using other temporal modeling approaches. Furthermore, as advanced disease-surveillance systems grow to monitor an increasing number of data streams, the risk of information overload increases. To address this problem, attempts to integrate information from multiple data streams have largely focused on detecting the multiple effects of a single outbreak across many data streams [28–31]. The approach described here is fundamentally different in that it focuses on detecting outbreaks in one data stream by monitoring fluctuations in its relationships to the other data streams, although it can also be used for detecting outbreaks that affect multiple data streams. We recommend using the network approaches described here alongside current approaches to realize the complementary benefits of both.

These findings suggest areas for future investigation. There are inherent time lags among epidemiological data streams: for example, pediatric data have been found to lead adult data in respiratory visits [32]. While the approach described here may implicitly model these relative time lags, future approaches can include explicit modeling of relative temporal relationships among data streams. It is also possible to develop this method further to track outbreaks in multiple hospitals and syndrome groups. It is further possible to study the effects on timeliness of detection of different network approaches. Also, while we show the utility of the network approach for monitoring disease patterns on a regional basis, networks constructed from national or global data may help reveal important trends at wider scales.

## Abbreviations

AEGIS: Automated Epidemiological Geotemporal Integrated Surveillance
CUSUM: cumulative sum
EWMA: exponential weighted moving average
SARS: severe acute respiratory syndrome

## References

[pmed-0040210-b001] Longini IM, Nizam A, Xu S, Ungchusak K, Hanshaoworakul W (2005). Containing pandemic influenza at the source. Science.

[pmed-0040210-b002] Ferguson NM, Cummings DA, Cauchemez S, Fraser C, Riley S (2005). Strategies for containing an emerging influenza pandemic in Southeast Asia. Nature.

[pmed-0040210-b003] Brookmeyer R, Johnson E, Bollinger R (2004). Public health vaccination policies for containing an anthrax outbreak. Nature.

[pmed-0040210-b004] World Health Organization Writing Group (2006). Nonpharmaceutical interventions for pandemic influenza, national and community measures. Emerg Infect Dis.

[pmed-0040210-b005] Mills CE, Robins JM, Lipsitch M (2004). Transmissibility of 1918 pandemic influenza. Nature.

[pmed-0040210-b006] Miller B, Kassenborg H, Dunsmuir W, Griffith J, Hadidi M (2004). Syndromic surveillance for influenzalike illness in ambulatory care network. Emerg Infect Dis.

[pmed-0040210-b007] Schrag SJ, Brooks JT, Van Beneden C, Parashar UD, Griffin PM (2004). SARS surveillance during emergency public health response, United States, March-July 2003. Emerg Infect Dis.

[pmed-0040210-b008] Ferguson NM, Keeling MJ, Edmunds WJ, Gani R, Grenfell BT (2003). Planning for smallpox outbreaks. Nature.

[pmed-0040210-b009] Bravata DM, McDonald KM, Smith WM, Rydzak C, Szeto H (2004). Systematic review: Surveillance systems for early detection of bioterrorism-related diseases. Ann Intern Med.

[pmed-0040210-b010] Mandl KD, Overhage JM, Wagner MM, Lober WB, Sebastiani P (2004). Implementing syndromic surveillance: A practical guide informed by the early experience. J Am Med Inform Assoc.

[pmed-0040210-b011] Wagner MM, Tsui FC, Espino J, Hogan W, Hutman J (2004). National retail data monitor for public health surveillance. MMWR Morb Mortal Wkly Rep.

[pmed-0040210-b012] Hutwagner LC, Maloney EK, Bean NH, Slutsker L, Martin SM (1997). Using laboratory-based surveillance data for prevention: An algorithm for detecting Salmonella outbreaks. Emerg Infect Dis.

[pmed-0040210-b013] Lau JTF, Yang X, Pang E, Tsui HY, Wong E (2005). SARS-related perceptions in Hong Kong. Emerg Infect Dis.

[pmed-0040210-b014] Woodward G, Stukel T, Schull M, Gunraj N, Laupacis A (2004). Utilization of Ontario's health system during the 2003 SARS outbreak.

[pmed-0040210-b015] World Health Organization (2006). Pandemic influenza preparedness and mitigation in refugee and displaced populations. Programme on Disease Control in Humanitarian Emergencies Communicable Diseases Cluster.

[pmed-0040210-b016] Wetterhall SF, Coulombier DM, Herndon JM, Zaza S, Cantwell JD (1998). Medical care delivery at the 1996 Olympic Games. Centers for Disease Control and Prevention Olympics Surveillance Unit. JAMA.

[pmed-0040210-b017] Dafni UG, Tsiodras S, Panagiotakos D, Gkolfinopoulou K, Kouvatseas G (2004). Algorithm for statistical detection of peaks—Syndromic surveillance system for the Athens 2004 Olympic Games. MMWR Morb Mortal Wkly Rep.

[pmed-0040210-b018] Bradley CA, Rolka H, Walker D, Loonsk J (2005). BioSense: Implementation of a national early event detection and situational awareness system. MMWR Morb Mortal Wkly Rep.

[pmed-0040210-b019] Reis BY, Mandl KD (2003). Time series modeling for syndromic surveillance. BMC Med Inform Decis Mak.

[pmed-0040210-b020] Reis BY, Pagano M, Mandl KD (2003). Using temporal context to improve biosurveillance. Proc Natl Acad Sci U S A.

[pmed-0040210-b021] Mandl KD, Reis B, Cassa C (2004). Measuring outbreak-detection performance by using controlled feature set simulations. MMWR Morb Mortal Wkly Rep.

[pmed-0040210-b022] Sartwell PE (1950). The distribution of incubation periods of infectious disease. Am J Epidemiol.

[pmed-0040210-b023] Knight J (2003). Harvard team suggests route to better bioterror alerts. Nature.

[pmed-0040210-b024] Cooper DL, Verlander NQ, Smith GE, Charlett A, Gerard E (2006). Can syndromic surveillance data detect local outbreaks of communicable disease? A model using a historical cryptosporidiosis outbreak. Epidemiol Infect.

[pmed-0040210-b025] Kulldorff M, Heffernan R, Hartman J, Assunção R, Mostashari F (2005). A space–time permutation scan statistic for disease outbreak detection. PLoS Med.

[pmed-0040210-b026] Heffernan R, Mostashari F, Das D, Karpati A, Kulldorff M (2004). Syndromic surveillance in public health practice: The New York City emergency department system. Emerg Infect Dis.

[pmed-0040210-b027] Das D, Mostashari F, Weiss D, Balter S, Heffernan R (2004). Monitoring over-the-counter pharmacy sales for early outbreak detection in New York City. MMWR Morb Mortal Wkly Rep.

[pmed-0040210-b028] Buckeridge DL, Burkom H, Campbell M, Hogan WR, Moore AW (2005). Algorithms for rapid outbreak detection: A research synthesis. J Biomed Inform.

[pmed-0040210-b029] Reis BY, Mandl KD (2003). Integrating syndromic surveillance data across multiple locations: Effects on outbreak detection performance. Proc AMIA Symp.

[pmed-0040210-b030] Burkom HS, Murphy S, Coberly J, Hurt-Mullen K (2005). Public health monitoring tools for multiple data streams. MMWR Morb Mortal Wkly Rep.

[pmed-0040210-b031] Ozonoff A, Forsberg L, Bonetti M, Pagano M (2004). Bivariate method for spatio-temporal syndromic surveillance. MMWR Morb Mortal Wkly Rep.

[pmed-0040210-b032] Brownstein JS, Kleinman KP, Mandl KD (2005). Identifying pediatric age groups for influenza vaccination using a real-time regional surveillance system. Am J Epidemiol.

